# Differential effects of wakeful rest, music and video game playing on working memory performance in the *n*-back task

**DOI:** 10.3389/fpsyg.2015.01683

**Published:** 2015-10-30

**Authors:** Maxim S. Kuschpel, Shuyan Liu, Daniel J. Schad, Stephan Heinzel, Andreas Heinz, Michael A. Rapp

**Affiliations:** ^1^Department of Psychiatry and Psychotherapy, Charité Universitätsmedizin Berlin (Campus Charité Mitte), Berlin, Germany; ^2^Social and Preventive Medicine, Universität Potsdam, Potsdam, Germany; ^3^Department of Psychology, Humboldt-Universität zu Berlin, Berlin, Germany

**Keywords:** break interventions, computer games, mozart effect, working memory, attention, cognitive resources, mind wandering

## Abstract

The interruption of learning processes by breaks filled with diverse activities is common in everyday life. We investigated the effects of active computer gaming and passive relaxation (rest and music) breaks on working memory performance. Young adults were exposed to breaks involving (i) eyes-open resting, (ii) listening to music and (iii) playing the video game “Angry Birds” before performing the *n*-back working memory task. Based on linear mixed-effects modeling, we found that playing the “Angry Birds” video game during a short learning break led to a decline in task performance over the course of the task as compared to eyes-open resting and listening to music, although overall task performance was not impaired. This effect was associated with high levels of daily mind wandering and low self-reported ability to concentrate. These findings indicate that video games can negatively affect working memory performance over time when played in between learning tasks. We suggest further investigation of these effects because of their relevance to everyday activity.

## Introduction

Every task can potentially be interrupted, preceded or followed by a break. Long-established break activities like wakeful resting and listening to music today are joined by more novel diversions like communicating on social media or playing computer games (Edlund, 2010). While most often we see breaks to provide a respite from task-induced fatigue, they may have an equally important role in setting up and modifying future task performance (Edlund, 2010). Here, we investigate the effects of different break activities on subsequent working memory performance.

Working memory is the retention of and operation on information over a short amount of time: its capacity is described by the ability to maintain information in the face of interference (Baddeley, 1992). Current theories of working memory involve executive control processes, which mediate the distribution of cognitive resources. This process can depend on lowered attentional control, which has been described as a mechanism in the context of “mind wandering” (Smallwood and Schooler, 2006). Our minds often wander during our daily activities, mind wandering thus filling up to 50% of our waking time (Killingsworth and Gilbert, 2010). Self-reported mind wandering was found to be systematically associated with particular contexts (Kane et al., 2007): subjects’ minds wandered more when they were tired or when they were involved in unpleasant activities and conversely wandered less when they were concentrated and when they were involved in enjoyable activities (Kane et al., 2007). This frequent mental activity occurs at a cost, being reportedly associated with reduced task performance (Mooneyham and Schooler, 2013), decreased attention (Allan Cheyne et al., 2009) and lowered working memory (Smallwood and Schooler, 2006; McVay and Kane, 2010). There are two theories that have been suggested regarding the relationship between mind wandering and measures of working memory (Smallwood and Schooler, 2006; McVay and Kane, 2010) suggested that mind wandering demands executive resources and reduces their availability for external information. McVay and Kane (2010), on the other hand, argued that mind wandering may result from executive-control failures. Smallwood (2013) argued that both hypotheses may describe different aspects of the initiation and maintenance of mind wandering. Recently, Thomson et al. (2015) proposed a novel “resource-control” theory to combine both “resource” (Smallwood and Schooler, 2006) and “control-failure” (McVay and Kane, 2010) theories of mind wandering to explain performance decrements over time. They suggested that as executive control fades over time, resource distribution favors mind wandering, which, in turn, decreases performance. Consistent with this view, Thomson et al. (2014) recently found that the cost of mind wandering not only decreases overall performance in a high-demand task overall but is also associated with a decrease in accuracy over time in both low- and high-demand tasks. This is consistent with the levels-of-inattention hypothesis (Schad et al., 2012) suggesting that increased time on task can not only induce mental shifts from task-performance to mind wandering, but can also alter the nature of the mind wandering state by eliciting deeper levels of mind wandering or inattention.

To assess the effect of everyday activities during a break on working memory performance, we chose three common break activities: Eyes-open resting was compared to listening to music and playing a video game. We specifically chose these three break activities in our study because they are often used in empirical studies and they are popular and widespread in everyday life (Jaušovec et al., 2006; Bavelier et al., 2011; Dewar et al., 2012).

Restful breaks have been found to be one of the most effective interventions connected to improving working memory performance (Ross et al., 2014; Helton and Russell, 2015). However, Lim et al. (2013) found that improvement of task performance after a break may also depend on individual differences in the ability to best make use of break opportunities. Resource theory implies that the ability of an intervention to afford performance recovery is higher if there is little overlap between the intervention and the specific processing resources of the primary task. Helton and Russell (2015) found that visuospatial task performance was best after a “complete rest” break in comparison to the four other break interventions, which consisted of either (1) continuing performance of the visuospatial task or alternately engagement with (2) an alphanumeric letter detection task, (3) a spatial memory task or (4) a verbal memory task.

Music has been linked to aspects of cognitive performance by the so called “Mozart Effect,” where short-term listening to classical music significantly enhanced spatial reasoning (Rauscher et al., 1993). However, this effect is highly controversial: More recent research suggests that beneficial effects of music are mediated by mood, arousal and musical preference, rather than being a discrete and unique effect of the musical qualities of classical music (Thompson et al., 2001; Koelsch, 2014).

Video games have been linked to enhancement in a wide variety of perceptual, attentional, and cognitive abilities (Boot et al., 2008; Eichenbaum et al., 2014). For example, expert video game players often outperform non-players on working memory tasks (Boot et al., 2008). Meanwhile, video games have been associated with a variety of negative outcomes: Gaming in general has been found to be able to induce physiological stress (Hébert et al., 2005), and the sound of a video game can disturb the players’ concentration (Lipscomb and Zehnder, 2004; Bavelier et al., 2011). The positive or negative effect of video game playing on cognitive performance can depend on the types of video games played (Barlett et al., 2009). For example, Boot et al. (2008) found that playing a highly complex strategy game can improve executive control and memory more than playing an action or a puzzle game. Playing violent video games, on the other hand, was shown to decrease the ability to exert executive control (Barlett et al., 2009). So far, research has rarely explored the effects of video games interleaved with working memory tasks, though a few studies have already found an effect on explicit memory performance (Dewar et al., 2012; Tang, 2013).

We expected gaming and music to have a differential effect on working memory performance compared to rest via their effects on mind wandering. We hypothesized that different breaks may have different effects on working memory function over time and we investigated a previous suggestion that such differences could be linked to mind wandering (Thomson et al., 2014).

## Materials and Methods

### Subjects

Thirty-five right-handed healthy native German subjects (18 female; age range: 19–32, Mean = 24.51, SD = 3.42) were recruited through advertisements in Berlin. Subjects were screened for major psychiatric disorders (SCID-I screening questionnaire) and underwent neuropsychological testing including verbal knowledge (Lehrl, 2005), fluid intelligence and cognitive speed (Wechsler, 1997), memory and executive functioning (Army Individual Test Battery, 1944; Isaacs and Kennie, 1973; Morris et al., 1989; Wechsler, 1997; Table 1). Social and demographical data, video gaming experience (time per week and types of games played) and music listening habits (time per week, types of music listened to) were gathered. Their daily mind wandering (DMW) was assessed based on the self-report Mind-Wandering Questionnaire (MWQ; Mrazek et al., 2013). We measured these variables and also assessed individual differences in cognitive abilities (e.g., fluid and crystallized intelligence, working memory and habits), which have previously been shown to influence memory performance (Lezak et al., 2012) to control for potentially confounding variables (see exploratory data analysis in Results section). Subjects were given detailed information and provided fully informed written consent. The study was approved by the Ethics Committee of Charité—University Medicine Berlin and was performed in accordance with the ethical standards laid down in the 1964 Declaration of Helsinki.

**TABLE 1:** **Sociodemographic information, characteristics of subjects and neuropsychological battery test performance**.

****	***N* = 35**
Age (years)	24.51 (0.58^a^)
Education (years)	16.14 (0.43)
Time spent on listening to music per week (hour)	10.32 (1.53)
Time spent on gaming per week (hour)	3.19 (1.20)
Fluid intelligence cognitive speed (DSST)	87.03 (1.62)
Verbal knowledge (MWT-B)	27.31 (0.59)
Verbal memory (Wordlist)	9.11 (0.19)
Verbal working memory (DS)	7.40 (0.30)
Semantic verbal fluency (SVF)	29.49 (0.99)
Executive functioning (TMT-A, seconds)	27.66 (1.60)
Executive functioning (TMT-B, seconds)	54.66 (3.01)
Daily mind wandering score (*N* = 32)	3.44 (0.14)
^a^Standard error of the mean (SEM)	

Cognitive Speed was assessed by the Digit Symbol Substitution Test (DSST) from the WAIS-R (Wechsler, 1997); Verbal Knowledge was assessed by the German Vocabulary Test (Mehrfachwahl-Wortschatz-Intelligenztest, MWT-B; Lehrl, 2005); Verbal memory was assessed by Wordlist from the Consortium to Establish a Registry for Alzheimer’s Disease (CERAD; Morris et al., 1989); Verbal Working Memory was assessed by the Digit Span (DS) Backward Test (Wechsler, 1997); 1-min Semantic Verbal Fluency (SVF) tested for the category “animals” (Verbale Flüssigkeit Tiere; Isaacs and Kennie, 1973); Executive Functioning was assessed by the Trail Making Test (TMT-A, TMT-B; Army Individual Test Battery, 1944); Subjects daily mind wandering was assessed based on the self-report Mind-Wandering Questionnaire (MWQ; Mrazek et al., 2013).

### Break Activity Scenarios

To evaluate the effects of different break activities on working memory, subjects were instructed to engage in “eyes-open resting” (rest quietly with their eyes open), “listening to music” (Mozarts “Sonata for Two Pianos in D Major, KV. 448—Allegro con spirito” over headphones) and “playing a video game” (play the “Angry Birds” video game, Rovio Entertainment, 2013, on a laptop computer) during an 8:30 min break following the training phase of the *n*-back task. The training phase consisted of two blocks of 2-back and two blocks of 3-back tasks. In the literature, ranges from 5 to 20 min have been reported for break duration (Travis, 1937; Dewar et al., 2012); our 8:30 min break duration was based on the length of the piece of music and was well within the range of these previous studies. Mozarts Sonata KV.448 has been a major musical piece in previous investigations of the effects of music on cognitive functions (Thompson et al., 2001; Rauscher, 2002; Kim and Lee, 2013). Angry Birds is a popular casual game, which utilizes concepts of spatial representation and has also been previously used in research (Ferreira et al., 2013; Kim and Lee, 2013). Both have been associated to spatial reasoning and memory performance (Thompson et al., 2001; Rauscher, 2002; Kim and Lee, 2013).

### Procedure

**FIGURE 1:**
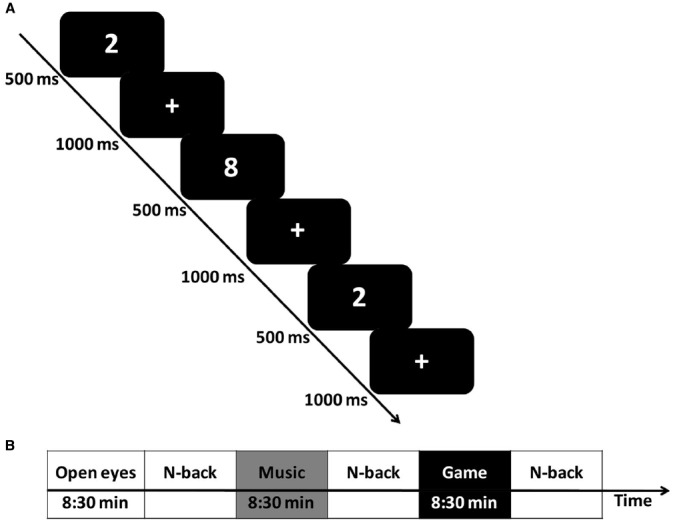
**(A)** *N*-back task (example: 2-back). White numbers were presented on a black background for 500 ms each, followed by a white fixation cross. The length of the interstimulus interval (ISI) was 1000 ms. **(B)** Testing procedure.

The general procedure is depicted in Figure 1. We utilized the classical *n*-back task (Cohen et al., 1994) implemented via Presentation^®^ software (Version 10.81, 2004, Neurobehavioral Systems Inc., Albany, CA, USA). In the *n*-back task, digits from 0 to 9 were visually presented in the center of an otherwise black screen in a randomized sequence one at a time as previously published (Heinzel et al., 2014a,b,c). Only two memory loads (2- and 3-back) were used because strong ceiling effects were found in younger adults at 0- and 1-back (Heinzel et al., 2014a,b,c). In the 2-back condition, subjects were required to press a response button if the current stimulus was identical to the stimulus presented two trials ago (see Figure 1). In the 3-back condition, subjects had to match the stimulus presented 3 trials ago. Stimulus duration was set to 500 ms, while the interval between stimuli (inter stimulus interval) was 1000 ms. Subjects performed 12 blocks of *n*-back task (randomly alternating between 2- and 3-back) with six blocks of each memory load condition. Each *n*-back block consisted of 20 trials with five targets (25% of trials).

All subjects began with a two block training session that consisted of 20 trials for each memory load (2- and 3-back, inter stimulus interval = 1500 ms). Immediately after training, subjects engaged in an 8:30 min break of either eyes-open resting, listening to music or gaming. Afterward, they started the main task. A repeated measures design was used with within-subjects factors for the three break activities (rest vs. music vs. game). The presentation order of the two different versions of the *n*-back task was randomized across blocks and the order of the three break activities were counterbalanced across subjects with a latin square. Subjects were randomly assigned to one of the presentation- and break orders^1^.

We used subjects’ self-reported DMW score (Mrazek et al., 2013) to assess mind wandering in every day life. The MWQ is a face-valid tool for rapidly assessing the levels of mind-wandering (Mrazek et al., 2013). In addition, in order to assess subjects’ mental activity during the task without interrupting their engagement with the break activities and *n*-back task, we followed previous studies (Gruberger et al., 2013) that used visual analogue scales (VAS; Bond and Lader, 1974) after the task as self-report measures. Immediately after every *n*-back task, we asked subjects to mark on a 100 mm straight line “Your ability to concentrate on the *n*-back task” from “Not concentrated at all” to “Very concentrated,” and also on a 100 mm line “The extent to which you thought about the *n*-back task during the break” from “Did not think about it at all” to “Thought about it all the time.” Responses were quantified as a score indicating distance, from 0 to 100 measured in millimeters, from the “Not at all” to the subject’s mark, with higher scores indicating greater ability to concentrate on the *n*-back task and higher mind wandering during the break. VAS measures have shown acceptable reliability and validity (Bond and Lader, 1974). Previous research found that self-reported inability to concentrate on the task assessed via VAS was associated with task performance deficits (Wearden and Appleby, 1997). Low level of task unrelated thought, self-reported via VAS, were interpreted as a possible index of focused engagement on the task (Gruberger et al., 2013).

### Analysis Using Mixed-effects Models and Models Comparison

We used the R system for statistical computing, *version 3.1.0*^2^ for data analysis. We estimated multilevel regression models using the *lme4* linear mixed effects package (Bates et al., 2014). We constructed a linear mixed-effects model to evaluate whether different break activities had an influence on overall memory performance and on performance changes over time. The data was averaged within each of six blocks of consecutive trials (each containing 20 trials). The difference between hit and false alarm rate was used as the dependent variable for these analyses.

For overall memory performance, we used the predictors *memory load* (2- vs. 3-back; effect coding: +0.5 vs. –0.5) and *break activity* (sliding differences contrast: rest vs. music, game vs. rest; using the MASS package (Venables and Ripley, 2002) and R-function *contr.sdif*) as well as their interaction as fixed effects. In addition, we included random subject intercepts, random subject slopes for the main effects, as well as random effects correlations. Furthermore, we tested whether additional random subject slopes for the interaction were justified and if there was any improvement in model fit (based on log-likelihood ratios, the Akaike information criterion (AIC; Akaike, 1977) or the Bayesian Information Criterion (BIC; Schwarz, 1978). For statistical tests of fixed effects parameters, we used the Satterthwaite approximation implemented in the *lmerTest* package (Kuznetsova et al., 2015).

For analyzing the performance over the course of the task, we used the same analysis as for the overall memory performance, except that we added the predictor *block* (via a linear effect for blocks 1 to 6 [mean-centered]) as a fixed effect as well as random slopes and correlations for the block-effect and tested the effects of *block* and *break activity* in the 2- and 3-back tasks separately. Furthermore, we investigated the influence of the test order of the three break activities by adding one predictor *break order* as the main effect, as well as its interactions with *block*, *break activity*, and their interactions (two-tailed significance testing was assumed). Additionally, we explored the effects of the potentially confounding individual neuropsychological test scores and habits (music/gaming) on individual subject estimates for the differences between rest versus music conditions and game versus rest conditions. To this end we performed individual regression analyses for each subject. We then tested if individual subject estimates (i.e., β) for the differences between rest versus music conditions and game versus rest conditions were correlated with individual neuropsychological test scores and habits (music/gaming). Significance values were set at *p* < 0.006 after Bonferroni correction for nine statistical comparisons.

To describe subjects’ self-reported ability to concentrate on the task after the break and the extent to which they thought about the task during the break, we used a repeated-measures ANOVA with the within-subjects factors break activity (rest vs. music vs. game). When the assumption of sphericity was violated, degrees of freedom were corrected using Greenhouse-Geisser estimates. Paired *t*-tests were used for pairwise comparisons and significance values set at *p* < 0.02 after Bonferroni correction for three statistical comparisons.

Finally, to investigate the effects of mind wandering and self-reported ability to concentrate on break-induced changes in memory performance, the average score of the self-report MWQ and the two VAS questions were added in the best fitting model. We tested the influence of each variable separately by adding one predictor as a main effect as well as its interactions with *block*, *break activity*, and their interaction (two-tailed significance testing was assumed). Furthermore, we tested if individual neuropsychological test scores correlated either with DMW scores, or with subjects’ self-reported ability to concentrate on the task after three break conditions.

## Results

### Group Description

Sociodemographic information, characteristics of subjects and results from the neuropsychological battery are presented in Table 1. On average, subjects spent 7.1 h per week more on listening to music than on gaming (averaging 3.2 h/week) [*t*(34) = –4.62, *p* < 0.001]. Among the 35 subjects, there were four frequent gamers, who preferred to play strategy video games (according to the definition, by Kühn and Gallinat, 2014), and no professional musicians.

### Behavioral Ratings

**FIGURE 2:**
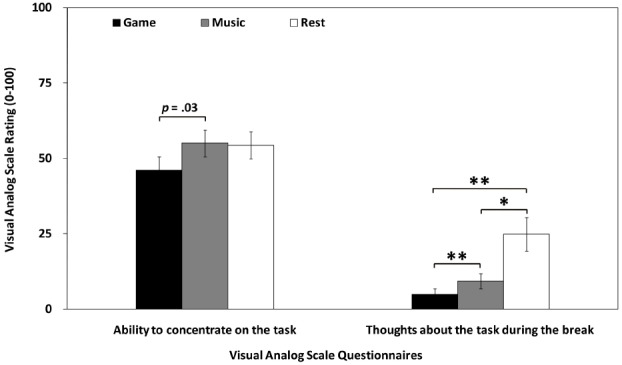
**Mean self-rated visual analog scores for game, music and rest conditions.** Visual analog scales (VAS) were adapted from Bond and Lader (1974). Error bars represent standard errors of the mean. *p < 0.02 and **p < 0.003 (two-tailed) after Bonferroni correction for multiple comparisons.

For the VAS ratings, we found a trend-wise effect of the ability to concentrate on the task [*F*(2, 68) = 3.10, *p* = 0.05]: Subjects’ self-rated concentration on the task was 9% better after listening to music (Mean = 55.00%, SE = 4.43%) as compared to after playing the video game (Mean = 46.11%, SE = 4.43%), shown in Figure 2 [*t*(34) = –2.35, *p* = 0.03]. The ability to concentrate on the task after eyes-open resting (Mean = 54.31%, SE = 4.43%) was not significantly different from listening to music or gaming (both *p*s > 0.07). We also observed a significant effect in the extent to which subjects thought about the task while engaging in different break activities [*F*(1.1,32.4) = 9.94, *p* = 0.003, Greenhouse-Geisser correction]. Paired *t*-test showed that during eyes-open resting (Mean = 24.84%, SE = 5.57%), subjects thought 20% and 16% more about the task as compared to gaming [*t*(31) = 3.47, *p* = 0.002] and listening to music [*t*(31) = 2.75, *p* = 0.01], respectively, shown in Figure 2. Moreover, subjects thought 5% more about the task [*t*(30) = 3.35, *p* = 0.002] during listening to music (Mean = 9.35%, SE = 2.47%) as compared to gaming (Mean = 4.81%, SE = 1.99%), shown in Figure 2.

#### Overall Working Memory Performance

In our analysis of the influence of break activities on overall memory performance, the data of all 35 subjects was best fitted by a model where the effects of *memory load* (2- vs. 3-back) and *break activity*, as well as their interactions were estimated as fixed effects and random subject intercepts and slopes for the two main effects as well as random effects correlations were estimated. Compared to this model, additional random slopes for the interactions between main effects gave no reliable improvement in model fit (all *ps* > 0.71). In the best fitting model of subjects’ task performance, the main effect of the first *break activity* contrast (rest vs. music) showed that subjects performed the task marginally better after eyes-open resting than after listening to music, β = 2.7, *t* = 1.72, *p* = 0.09. However, overall performance after gaming did not significantly differ neither from eyes-open resting, β = –2.4, *t* = –1.08, *p* = 0.29, reflected in the second *break activity* contrast (game vs. rest), nor from listening to music, β = 0.4, *t* = 0.19, *p* = 0.85, shown in a *post hoc* test contrasting game vs. music. Moreover, there was no significant *memory load* × *break activity* interaction (both *p*s > 0.64).

### Time Course of Working Memory Performance

Next, we investigated whether different break activities had differential effects over the time course of the working memory task. We modeled the task performance data using the fixed effects *block* (*n*-back block 1 to 6; linear coding), *break activity* (sliding differences contrast: rest vs. music, game vs. rest), and their interactions, random intercepts and random slopes for the two main effects, and random effects correlations. Additional random slopes for the interactions between main effects gave no reliable improvement in model fit [χ^2^_(28)_ = 6.57, *p* > 0.83, logLik_0_ = –12, logLik_1_ = –9; AIC_0_ = 58, AIC_1_ = 73; BIC_0_ = 133, BIC_1_ = 198], and were omitted from the model.

**FIGURE 3:**
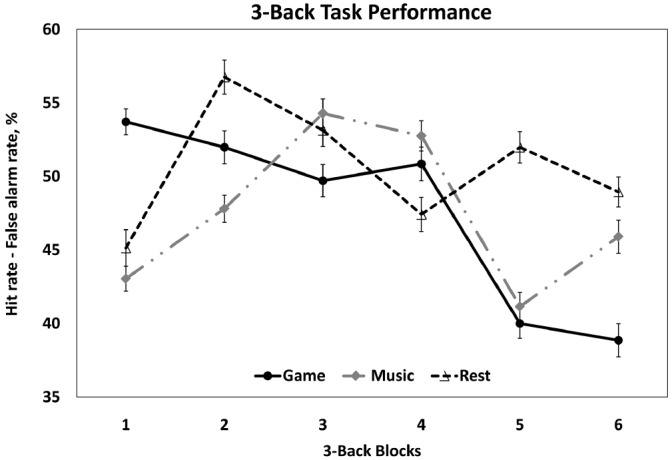
**Mean task performance in ***3-back*** as a function of block (***n***-back block 1 to 6) and break activity (game vs. music vs. rest).** Error bars represent standard errors of the mean.

Based on this model of task performance in the *3-back* task, we did not find differences in subjects’ task performance over time after eyes-open resting to be different as compared to listening to music (β = 0.2, *t* = 0.14, *p* = 0.89). However, we found a significant interaction between *block* and *break activity* (game vs. rest), β = –3.1, *t* = –2.33, *p* = 0.02, in that after video gaming, subjects’ task performance was gradually decreasing over the course of the task as compared to eyes-open resting (Table 2, see also in Figure 3). *Post hoc* tests contrasting game vs. music showed that performance after gaming was also decreasing over time as compared to listening to music, β = –2.9, *t* = –2.20, *p* = 0.03. Furthermore, we found no significant three-way interaction between *block*, *break activity*, and *break order* (*p* = 0.22), indicating that there was no significant influence of the test *break order* on the observed break effects on task performance over time.

**TABLE 2:** **Model parameters of target sensitivity in the *3-back task***.

**Fixed effects parameter**	**Estimate**	**SE**	***t* value**	***p*^a^**
Intercept	48.5	2.2	21.80	<0.0001***
Block	–1.1	0.6	–1.88	0.06
Break activity (rest vs. music)	3.1	2.4	1.29	0.20
Break activity (game vs. rest)	–3.0	3.0	–1.00	0.32
Block × break activity (rest vs. music)	0.2	1.3	0.14	0.89
Block × break activity (game vs. rest)	–3.1	1.3	–2.33	0.02*
**Random Effects Parameter**			**Correlations**
**Group: Subjects**	**SD**	**Intercept**	**Block**	**Break activity (rest vs. music)**
Intercept	12.0			
Block	1.5	0.94		
Break activity (rest vs. music)	4.6	0.66	0.36	
Break activity (game vs. rest)	12.1	–0.03	0.32	–0.77
Residual	23.2			

^a^The lmerTest package was used to compute approximate p-values. *p < 0.05; **p < 0.01; ***p < 0.001 (two-tailed). Number of observations: 630, groups: 35. Significant results are marked bold.

Break activity did not affect variations in task performance over time in the *2-back* task (all *p*s > 0.21). We found no significant correlations of the individual neuropsychological test scores and habits (music/gaming), neither with respect to the estimates of individual differences between rest versus music conditions (all *p*s > 0.06, correlation with TMT-B: *p* = 0.06, *r* = –0.33), nor with game versus rest conditions (all *p*s > 0.07 except for a correlation with MWT_B: *p* = 0.02, *r* = 0.40), with significance values set at *p* < 0.006 after Bonferroni correction for nine statistical comparisons.

### Effects of Daily Mind Wandering and Self-reported Ability to Concentrate

**FIGURE 4:**
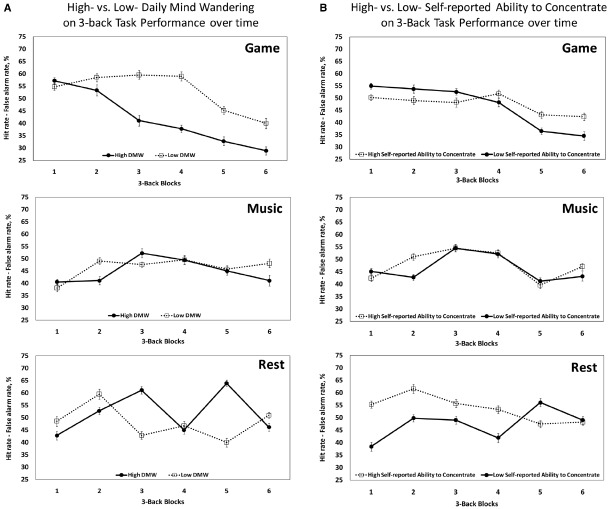
**The influence of daily mind wandering and self-reported ability to concentrate on ***3-back*** task performance over six blocks, after gaming, listening to music or eyes-open resting. (A)** The effect of high- (*N* = 12) vs. low- (*N* = 14) daily mind wandering (DMW) scores on *3-back* task performance after game, music and rest, respectively. Subjects’ self-reported DMW score was ranked higher vs. lower than median (*N* = 26/35: six subjects’ data in the median was removed). **(B)** The effect of high- (*N* = 17) vs. low- self-reported ability to concentrate (*N* = 17) scores on *3-back* task performance after game, music and rest, respectively. Subjects’ self-reported ability to concentrate (i.e., the ability to concentrate on the task after the three break conditions), was ranked higher vs. lower than median (*N* = 34/35: one subject’s data, in the median was removed).

To investigate the effects of mind wandering, we tested if the self-report DMW questionnaire and the two VAS questions for self-reported ability to concentrate (i.e., the ability to concentrate during the task and thoughts about the task during the break) would contribute to the significant interaction between *block* and *break activity* (game vs. rest) in the *3-back* task performance. We found a significant three-way interaction between *break activity* (game vs. rest), *block* and self-reported DMW, β = –2.7, *t* = –1.96, *p* = 0.05, showing that the impairment of task performance over time after gaming was particularly prominent in subjects scoring high in DMW (*p* = 0.002), but was not significant in those scoring low in DMW (*p* = 0.06; see in Figure 4A). Similarly, we found a significant three-way interaction involving subjects’ ability to concentrate on the task after the break, β = 3.6, *t* = 2.7, *p* = 0.007, indicating a larger decline of task performance over time after gaming in less concentrated subjects (*p* = 0.02; see Figure 4B). However, the respective interaction involving the extent to which subjects thought about the task during the break was not significant, β = –1.8, *t* = –0.81, *p* = 0.42, indicating that thinking about the *n*-back task during the break did not significantly influence performance on the *n*-back task.

Furthermore, exploratory data analysis showed that the neuropsychological test scores did neither correlate significantly with DMW (all *p*s > 0.32, correlation with SVF: *p* = 0.32), nor with self-reported ability to concentrate (all *p*s > 0.12, correlation with SVF: *p* = 0.12).

Importantly, we also tested whether individual’s DMW was related to the self-reported ability to concentrate on the *n*-back task (measured via the VAS), and indeed found that higher levels of mind wandering were associated with lower self-reported ability to concentrate (Pearson *r* = –0.301, *p* < 0.05, one-tailed), suggesting that both measures tap into related aspects of mind wandering and self-reported ability to concentrate.

## Discussion

We investigated whether break activities used in daily life (i.e., wakeful resting, listening to music, and playing a video game) interact with working memory performance in young adults. We found that a short period of passive eyes-open resting trendwise enhanced overall task performance as compared to passively listening to music. Actively playing a video game, to the contrary, had no influence on overall task performance. Our main finding is a differential effect of active gaming as compared to passive eyes-open resting and listening to music on the time course of working memory performance, in that after gaming subjects showed a gradual decrease of the memory performance over the course of the task compared to the two other break conditions. Interestingly, this effect was associated with increased DMW and decreased self-reported ability to concentrate.

### Overall Task Performance is Better After Rest as Compared to Music

When looking at the overall effect of wakeful resting, listening to music and gaming, task performance after eyes-open resting was trendwise better compared to listening to music, hypothetically because subjects also thought more about the task during open eyes resting than during listening to music. Consistent with the resource allocation theory, our result partially supports the hypothesis that rest is the most efficient replenishment of a common executive resource (Helton and Russell, 2011, 2015). Importantly, however, effects of break activities on overall task performance were weak, and not significant. This observation suggests that break activities may not strongly affect overall working memory performance on the *n*-back task. Instead, working memory performance seems to be relatively robust against such break activities at a global level. Interestingly, however, we found more subtle changes of task-performance across the time course of the task.

### Gradual Performance Decline After Gaming

After subjects played the “Angry Birds” game, their task performance gradually declined over the course of the 3-back task, compared to task performance after eyes-open resting and listening to music. Video gaming has been found to be reliably associated with an enhancement of visual attention (Bavelier et al., 2011) and increased visual attention induced by gaming may have helped subjects to better maintain task-relevant information at the beginning of the *n*-back task. Thus subjects started out with a stronger task performance after gaming, which gradually decreased over time. In support of this interpretation, immediately after every *n-*back task session, subjects reported that subjectively they had less ability to concentrate on the task after playing a video game as compared to listening to music, shown in Figure 2. This may reflect a gradual depletion of cognitive resources that are necessary for working memory performance (Helton and Russell, 2011). Importantly, we found that this gradual performance decline after gaming was closely related to mind wandering: it was strongest in individuals with a high propensity to mind wander in their daily life, and in those individuals with low levels of self-reported ability to concentrate. This result is consistent with previous reports of enhanced frequency (Thomson et al., 2014) and deeper levels (Schad et al., 2012) of mind wandering after prolonged task execution and with the “resource-control” framework of mind wandering (Thomson et al., 2015), suggesting that executive control over the content of thoughts is impaired after prolonged task execution, leading to intrusions of task-unrelated thoughts. In the present study, mind wandering mediated a performance decline specifically after playing computer games, but not after resting or listening to music. Based on resource-control theory, computer gaming may effectively represent performance of an external task even during the break, involving continued engagement of executive resources and their continued depletion over time, leading to mind-wandering-induced performance deficits. Listening to music or resting, to the contrary, should effectively interrupt external task-performance, allowing thoughts to wander, and may thus support recovery of executive resources. In summary, the results show that mind wandering and reduced self-reported ability to concentrate may contribute to declining performance in high working memory load conditions after spending time with video games rather than music or rest during learning breaks.

It is possible that the effects of video gaming on the decrement of working memory performance over time are due to its influence on subjects’ emotional states. Differing from listening to music and eyes-open resting, the “Angry Birds” game is designed to use sensory and emotional features to draw players’ attention (Kim and Lee, 2013). For instance, subjects hear the “bang” and “laughing” sound every time when they hit the target during gaming. Such emotional cues might let subjects engage more in playing the “Angry Birds” game compared to other two break activities and also to the *n*-back task. Upon resumption of the *n*-back task after gaming, subjects may have felt somewhat bored and may have had difficulties to allocate their attention to the task. Choi et al. (2013) found that emotional state interacts with working memory performance. To support this interpretation of our current results, it would be necessary to measure emotional states during breaks in future studies.

As an interesting possibility, prolonged task execution and depletion of executive resources may not only increase the propensity for mind wandering, but may also lead to increasingly deeper levels of mind wandering (Schad et al., 2012) after playing computer games compared to resting or listening to music, and may cause the mind to wander more deeply. Future research assessing different levels of inattention (e.g., via the sustained attention to stimulus task; Schad et al., 2012) will be needed to investigate this possibility empirically.

In contrast to the 3-back task, we did not find significant differential learning effects of task performance in the less demanding 2-back task, which might be because in a young adult population, this 2-back task is approximately the level of difficulty that can be done rather well and that does not maximally strain available resources (Chooi and Thompson, 2012). Our and other studies have also encountered ceiling effects with the 2-back (Mylius et al., 2012). Future studies will have to detail performance over longer periods of time following different break activities.

### Limitations

The observed effects of breaks on the *n*-back working memory task were found in young, well-educated subjects. Other types of music or games, e.g., self-selected (Cassidy and Macdonald, 2009), may result in different findings. We aimed at maximizing the ecological validity of the break conditions. Thus, future research will be needed to pinpoint the precise mechanisms by which eyes-open resting, listening to music, and gaming might exert their effects on working memory via cognitive processes like depletion of resources or of cognitive control. In our study, we found no break effect on overall task performance, suggesting that *n*-back performance by itself was not significantly changed by different break activities. However, our findings that gaming leads to a continuous decrement in task performance, which was related to both distal and proximal measures of mind wandering, is consistent with previous research findings of continuous performance decline due to mind wandering (Thomson et al., 2014). The current conclusions must remain tentative due to the lack of an overall effect as well as our limited sample size and power to detect more subtle effects of mind wandering or self-reported ability to concentrate. For a more comprehensive understanding and vigorous testing of this issue, future studies are needed to illuminate the nature of the interplay between break activities, mind wandering, and task performance. In order to not disturb subjects’ engagement with respective break activities, we have obtained the self-report measures of mind wandering and self-reported ability to concentrate retroactively. However, it may be preferable to use real-time experience sampling, such as the thinking content scale of the Dundee Stress State Questionnaire (DSSQ; Matthews et al., 2002), self-catch task-unrelated thought and intermittent thought probes (Smallwood and Schooler, 2015) during breaks and the *n*-back task. We also did not use questionnaires to evaluate subjects’ emotional state by considering both emotional arousal and valence at the same time (Choi et al., 2013). Contributions of emotional state will have to be validated by reliable physiological and behavioral data in future studies, which could also include measures of physiology (e.g., heart rate and skin conductance).

## Conclusion

We examined whether typical activities that people engage in during a break (i.e., eyes-open resting, listening to music and playing a video game) interact with working memory. We found that playing the Angry Birds video game before the *n*-back task reduced learning over time as compared to eyes-open resting and listening to Mozart’s music. Understanding working memory mechanisms during break activities may help to guide further research into optimal resting methods, including the usage of video gaming in young adults who try to relax between challenging tasks.

## Author Contributions

Conceived and designed the experiments: MSK, SL, DJS, SH, AH, MAR. Programed the experiments: SH Performed the experiments: MSK, SL. Analyzed the data: MSK, SL, DJS. Wrote the paper: MSK, SL, DJS, SH, AH, MAR.

### Conflict of Interest Statement

The authors declare that the research was conducted in the absence of any commercial or financial relationships that could be construed as a potential conflict of interest.

## Footnotes

1 www.randomizer.org

2 www.r-project.org
